# Mental Trauma Experienced by Caregivers of patients with Diffuse Axonal Injury or Severe Traumatic Brain Injury

**DOI:** 10.5812/traumamon.11522

**Published:** 2013-08-11

**Authors:** Syed Tajuddin Syed Hassan, Husna Jamaludin, Rosna Abd Raman, Haliza Mohd Riji, Khaw Wan Fei

**Affiliations:** 1 Department of Medicine, University of Putra Malaysia, UPM Serdang, Selangor, Malaysia; 2 Department of Computing Sciences, University of Teknologi MARA, Shah Alam, Selangor, Malaysia; 3 Department of Community Health, University of Putra Malaysia, UPM Serdang, Selangor, Malaysia

**Keywords:** Wounds and Injuries, Brain Injuries, Rehabilitation, Diffuse Axonal Injury

## Abstract

**Context:**

As with care giving and rehabilitation in chronic illnesses, the concern with traumatic brain injury (TBI), particularly with diffuse axonal injury (DAI), is that the caregivers are so overwhelmingly involved in caring and rehabilitation of the victim that in the process they become traumatized themselves. This review intends to shed light on the hidden and silent trauma sustained by the caregivers of severe brain injury survivors. Motor vehicle accident (MVA) is the highest contributor of TBI or DAI. The essence of trauma is the infliction of pain and suffering and having to bear the pain (i.e. by the TBI survivor) and the burden of having to take care and manage and rehabilitate the TBI survivor (i.e. by the TBI caregiver). Moreover many caregivers are not trained for their care giving task, thus compounding the stress of care giving and rehabilitating patients. Most research on TBI including DAI, focus on the survivors and not on the caregivers. TBI injury and its effects and impacts remain the core question of most studies, which are largely based on the quantitative approach.

**Evidence Acquisition:**

Qualitative research can better assess human sufferings such as in the case of DAI trauma. While quantitative research can measure many psychometric parameters to assess some aspects of trauma conditions, qualitative research is able to fully reveal the meaning, ramification and experience of TBI trauma. Both care giving and rehabilitation are overwhelmingly demanding; hence , they may complicate the caregivers’ stress. However, some positive outcomes also exist.

**Results:**

Caregivers involved in caring and rehabilitation of TBI victims may become mentally traumatized. Posttraumatic recovery of the TBI survivor can enhance the entire family’s closeness and bonding as well as improve the mental status of the caregiver.

**Conclusions:**

A long-term longitudinal study encompassing integrated research is needed to fully understand the traumatic experiences of caregivers. Unless research on TBI or DAI trauma is given its proper attention, the burden of trauma and injury on societies will continue to exacerbate globally.

## 1. Context

Dante Alighieri states: “I was not dead yet, but little of life remains.”A scourge of modern living is a silent trauma spreading fast in many countries. Worldwide, trauma is now the leading cause of death and disability for the under 45 year-old population (1, 2). By 2020 trauma due to motor vehicle accidents (MVA) will be the third largest global health burden. These result from at least 50 million injuries each year (2). Needless to say, the mental impact is traumatic as well and much more on the caregivers ( usually the victim’s family) (3, 4). The major focus of managers of MVA trauma have been mainly on the survivors/victims, not on the caregivers. There are hardly any long-term definitive studies on caring constructs such as compassion fatigue and burden “burnt-out” attitudes that relate to the continual and continuous low-feeling and suffering of the caregivers. 

Nevertheless, the caregivers’ experience is closely interlinked with that of the care-receivers. Obviously, the psychological pain is felt much more by the poor sectors of the world population, due to lack of essential resources for care giving and rehabilitation (1). The essence of trauma is the infliction of pain and suffering. It is associated with burden of having to bear the pain (i.e. TBI survivor), and burden of having to care and manage and rehabilitate the TBI survivor (i.e. TBI caregiver). Thus, in one sense, trauma applies even for mundane everyday life situations which inflict substantial mental pain, distress, and stress. Nevertheless in extraordinary intense pain (5) and stress situation involving physical, mental and psychological afflictions, trauma of care-receivers and caregivers associated with severe TBI patients, such as diffuse axonal injury (DAI), is paramount. In this short review on trauma related to TBI especially DAI, care giving is conceived as the process of intensively helping or providing assistance above and beyond the normal assistance usually given to family members and friends (6). It is associated with a high traumatic stress due to the intensity of care provided at all times, and the unending high levels of adverse sequelae of cognitive, emotional, memory, physical, behavioral, sexual, social and spiritual impairments and disability of the care-receivers (7). Rehabilitation which involves “repairing” and restitution of physical, spiritual and functional entities is a highly demanding and overwhelming task. Care giving involves doing tasks in caring and maintaining daily activities of living which in turn heals or rehabilitates the care-receiver. Thus, in the case of severe TBI or DAI survivor, who requires perpetual continuous caring similar to many highly chronic cases, the care giving job itself is highly demanding. Hence the ensuing trauma is an outcome of DAI affecting both victims and caregivers (8). The connections and interactions between the former and the latter involve many dimensions and determinants, and are critical since they subsequently determine the severity of trauma. The analogy used here is that of a journey the caregivers have to travel. It is a healing journey, for which most caregivers were not pre-prepared or pre-trained in advance (8). It is known that there is differential perception and acceptance of the inflicted TBI injury, its sequelae, and the consequential trauma, by different caregivers and care-receivers. To understand fully the relationship between care giving and care-receiving, a holistic or ecological approach should be solicited (9). Such a study is feasible since the rehabilitation process for severe brain injury can extend a lifetime. For severe TBI such as DAI, full recovery is seldom if ever achieved. In fact, it is well acknowledged that severe TBI survivors (a small fraction of total severe TBI or DAI victims- approximately 10%) continue to bear multiple long-term functional disorders and deficits, debilitating neurobehavioral sequelae, and persistent neuropsychological impairment (2). This review intends to highlight the hidden silent mental trauma experienced by the caregivers of severe brain injury survivors. 

## 2. Evidence Acquisition

### 2.1. Care Giving and Rehabilitation

Many victims of TBI, especially DAI, die during or subsequent to the accident. It is the survivors and their caregivers who are subjected to intense and pervasive trauma. Research on trauma in relation to TBI is dismal in the developing and poor countries. This short review alludes to TBI trauma challenges and issues, in these countries. Yet research findings on TBI trauma have to be drawn and inferred from the developed world. It is seen that researchers in developed countries are treating care giving and rehabilitation separately. Nevertheless, in actual cases, many aspects of care giving contribute towards rehabilitation (7). As shown in the literature, the caring and healing tasks are perpetually unnerving with unpredictable consequences and outcomes (10). It is common professional knowledge that no two brain injuries and their outcomes are exactly the same. It thus makes sense that any incremental success or outcome from care giving and rehabilitation acts to enhance the quality of life of both the caregivers (usually family or parents) and the care-receivers (TBI victims). Consequently care giving and rehabilitating are inseparable entities. Such an intractable relationship of care giving and rehabilitation has never been considered or emphasized before. Despite the body of research on injury, impairment and interventional aspects of TBI (10), research on the ensuing trauma is relatively scarce and neglected, even in the developed world. In terms of philosophy of mind, the feelings aspects of trauma should be at the forefront (11). It is clear that care giving and rehabilitation of TBI will contribute towards structuring of an overwhelmingly challenging and daunting lifelong chronic trauma for the caregiver and care-receiver. In time, the trauma deteriorates with asynchronous or dysfunctional relationship between the care giving task and the rehabilitation demand. Hence, long-term in-depth studies are required to clarify and quantify such critical and intractable relationships.

## 3. Results

### 3.1. Trauma of Care giving and Rehabilitation

Drastic experiences which inflict pain and unnerving frightful sensation of lasting memory comprise trauma, which can be physical, psychological and/or spiritual. Spiritual trauma has been less studied, yet it holds life defining significance for those who believe in religions. Logically, a traumatic experience should be defined and measured within the context of characteristics of a society, which is imbued with specific socio-cultural beliefs, political and ecological perspectives (11, 12). Nevertheless there are certain traumas with universal values which transcend societies, ethnicities and geographical boundaries. Trauma due to brain injury such as severe TBI and DAI is one such experience with universally accepted and similarly affected effects and impacts. Due to complexity of effects and impacts affecting the caregivers and care-receivers (TBI survivor), the trauma can be considered as complex trauma or poly-trauma. Thus trauma such as TBI or DAI has a multidimensional perspective embodying an “ecological system” of its own, with unique recovery or rehabilitation contexts (12). Affliction of severe brain injury redefines the meaning of life and reassigning the pathways of present and future life style and living environment of the entire family. Disruption of life’s normalcy signifies ominously the ensuing trauma of the affected care giving family. In addition to life’s disruption, many challenges confront a caregiving family. These include those that cater to the TBI survivor e.g. constant need to provide positive, stimulative, and innovative healing environment, and those of the caregiver e.g. ensuring accessibility and sufficiency of resources (financial, information, medication, etc.). All these demands add to the traumatic stress of the caregivers. It is almost a cliché that developed countries with their implicit materialistic culture and setting, are actually more responsive to humane needs and exigencies. Hence, especially in developed countries, with availability of care and resource allocation for the lucky few, the normalcy disruption can be morphed into life-functioning transformation, adaptation, and possibly even into a triumphant achievement, such as the posttraumatic growth, of trauma victims (6). It is widely acknowledged that a severely brain injured person is a changed human being. He/she is not his/her old self anymore. Consequently his/her thinking, psyche, behavior, and personality and demeanor change dramatically, completely or partially. These personality and behavior changes are further worsened by impairments due to sensory and neurological deficits, and cognitive difficulties and dysfunctions. Consequently, it is common knowledge that the care-receiver becomes unreasonably critical of the caregiver and vice-versa, hence physical and mental abuses may be subsequently and consequently inflicted by the care-receiver on the caregiver and vice-versa (10). In time, such abuses add to the negative burden and trauma dynamics of TBI or DAI caregiving and rehabilitation. The long term implication of such a scenario portrays a non-ending or exacerbating trauma setting for caregivers and care-receivers. Research on such a long term trauma setting is badly lacking, even in developed countries. It is well known too that many caregivers are not properly trained for their caregiving task (8), and not every caregiver can easily access pertinent information and guidance on caregiving from sources such as the media (internet etc.). Such defects occur even in developed countries, and more so in the developing and poor nations. These deficiencies thus compound the burden of trauma of caregivers in discharging their caregiving and rehabilitation assignments. Nevertheless, in Japan, positive aspects of TBI family care giving experiences indicated successful psychosocial adjustment, despite the developing and growing distress, due to successful adaptation to a transformational constructed system of care giving and rehabilitation, and social and informational empowerment (13). It is widely known that Japanese discipline, socio-cultural and value system highly upholds reverence towards family kinship and altruism. No similar report has been published with respect to any other society. Nevertheless, despite conflicting and contrasting impacts and effects, it is common knowledge that TBI caregivers’ mental trauma worsens in the elderly group i.e. with ageing. 

### 3.2. Qualitative Investigation

Assessment of human suffering such as DAI trauma can be reasonably accurately identified qualitatively even from a few detailed case studies. In contrast, direct physical symptoms such as post-traumatic stress disorder (PTSD) have been measured quantitatively, using physical scoring tools including those which evaluate memory fragmentation and accident-rumination (14). Evidently, many measurements are piece-meal perceptive evaluations, mostly from small sample size selection, and these do not reflect the magnitude of the total trauma experience throughout the care giving journey. Inevitably such measures identify parameters which at best portray a small window of the big picture. Examples of such measures are caregivers’ perceived burden, which influence rehabilitation outcome of the TBI victims, and caregivers’ self-rated resilience which directly correlates with adaptation in care giving (15). Moreover, due to high variability in methodology of many studies, only generic patterns and general logical results can be obtained (8). In contrast, detailed qualitative studies can illuminate and highlight stages or phases of life during the entire spatial-temporal spectrum of care giving and rehabilitation (16). Information from such studies can be value-added by providing illustrations such as visual models which depict relative magnitude of outcome components during the healing and recovery process(16). Perhaps innovative measures (17) can synthesize trauma scores into a multidimensional index. Trauma encompasses visible and invisible components, including an individual’s memory, thoughts, emotion, and actions (18). It is widely acknowledged that the lives of TBI victims and their families become transformed, hence transgressing the sacrosanct concept of completeness and wholeness of man as God’s creation (9). TBI affects quality of life of both care-receivers and caregivers, with majority of the care-receivers continuing to bear multiple functional disorders and deficits (19). Since the outcome of the process of care giving and rehabilitation is not precisely predictable, the art and science involved may be akin to Thomas Kuhn’s paradigm of subjectivity and volatility of scientific truth and progress. Thus truth, even about traumatic experience, may be considered as a societal construction rather than an emergent from accretion of empirical or positivism evidence. In essence, quantitative research focuses on characterizing, diagnosing, intervening, and prognosing for essentially medical treatment purposes. Qualitative research acts as an essential complement to unravel the full life journey and rhetorical narrating-dialoguing paradigms- the humane component (16). This can be done through invoking dynamic protocols of ontology, epistemology and methodology. Hence the holistic healing of trauma of both caregivers and care-receivers can be strategized and actualized.

### 3.3. Quality of Life

TBI is also considered as an acquired brain injury (ABI) (20). Another common form of ABI is stroke. Due to its worldwide pervasive occurrence and chronic persistence, there has been substantial research on stroke in terms of quality of life of victims and their respective caregivers. There are many similarities between stroke and TBI, particularly with respect to characteristics such as behavioral and self-awareness deficits, theory-of-mind dysfunctions (20,21), and related impairments such as alexithymia and lack of empathy. A most highlighted common deficit of ABI is the loss of self-identity, personality, anomy (21), self-integrity and in extreme cases sanity. Research is warranted on the two latter constructs. Published literature gives considerable focus on anxiety, coping, burden, depression, health, and resilience in relation to care giving(7, 8, 14, 15), but, with exception (22), offers little attention on positive aspects such as bonding, redeeming family kinship, and mutual learning between caregivers and care-receivers. Arango-Lasprilla et al. (23) have shown that in Guadalajara, Mexico (developing country) TBI caregivers suffered lower health-related quality of life (HRQoL) across many domains, including mental and general health. More research is necessary to elucidate which factors are critical in determining the HRQoL. It is common knowledge now that TBI survivors and their caregivers are also subjected to increasing social exclusion or abstinence. It is professionally recognized now that socialization or social support moderates coping of caregivers, hence their trauma experience. Such phenomena, in terms of the trauma, have not been well investigated. For the TBI survivor, it is well known also that his or her wellbeing is greatly affected and impacted by trauma due to impairments involving attention focus, memory difficulties, spatial orientation, loneliness, cognitive and intellectual entombment, and anomy. To this date most researches examine constructs which deal with anxiety, burden, coping, injury after-effects on care giving family functioning and resilience. There are a few ad hoc one-off qualitative studies and reviews e.g. those which described anxiety, needs and well-being of TBI caregivers and care-receivers (9, 12, 23). However, there are no published papers relating simultaneously to the trauma of both the caregivers and the TBI survivors; their interactive-integrative constructs and domains involved, interrelationship and dynamics, and functionalities of the processes of care giving and rehabilitation, and the humane qualitative aspects of the care giving-rehabilitating journey of the survivor and his/her caregivers. As alluded to by Ibn Sina for head injury trauma (24), a holistic approach to investigate TBI trauma (25) is warranted to enable the entire system to be elucidated, rather than ad hoc patchy or stand-alone studies. Hence integrated long-term researches should be the priority. An urgent need is for TBI to be legally declared as a disease, not as impairment. Only when this recognition occurs, can health authorities pay due attention to this silent epidemic.

## 4. Conclusions

There is substantial literature on TBI injury, on the survivors with respect to the concomitant and subsequent adverse sequelae of deficits, impairments, and disabilities (2). In contrast much less is written on the caregivers and their activities and efforts in daily caring and rehabilitating of the TBI survivors (care-receivers) (26). There has also been very little attention directed towards understanding and measuring the tangible and the intangible aspects of trauma experience and sensation (physical, psychological, spiritual) of the caregivers and the care-receivers. At present, it is known that even painful and distress experiences can be measured by psychometric scores, which estimate the extent and severity of the traumatic experience (13). Nevertheless the scoring system is still largely based on subjective evaluation of perceptions (27), and not on objective measures based on ecological, physiological entities, metabolic biochemistry, and on other biological mechanisms and processes. No meaningful analysis or synthesis has been made on long-term trauma experiences. It is evident that intensive research on the trauma aspects of TBI is urgently needed, in order to capture baseline information, so that strategies and tactics of interventions can be formulated. For TBI, the qualitative research approach in conceptualizing, evaluating and describing trauma of both the caregivers and care-receivers has only recently begun to be explored (6, 25). Much more research is needed using the qualitative approach so that true meanings of trauma experience can be extracted. Not surprisingly, most of earlier literature dwells on the negative aspects of outcome of care giving (26). It is apparent that the outcome is moderated by a number of interacting factors (7) which include family functioning adaptation, resilience, coping mechanism and style, community support, information empowerment (28), financial provision, spiritual/religious (28) enlightenment, and psychological adjustment and enhancement. As for the trauma experience, even those involving considerable physical aggression and self-injurious behavior of the TBI survivor (29), there is a well-validated evidence that indicates the contributory influence, role, and interaction effect of those factors or variables which can be well demarcated and explained through research using the qualitative approach (14). In TBI, healing of the thinking processes i.e. neurological restitution (30) and cognitive rehabilitation (31) predicates the subsequent intervention and consequential trauma (32). Thinking and cognition (the mind) define a person. Central to the trauma equation of the caregivers and the care-receivers, are the changed identity and personality, and the concomitant psyche and behavior of the TBI or DAI survivor (33). Hence in order to really understand and empathize physical and psychological trauma of TBI caregivers (34) and care-receivers (35), it is essential to institute conversations, capture and record their experience (including some quantitative measures such as assessing caregiver resilience (15)), analyze and assess to reveal the essence (including chronic pain (5)), render to fine-tune their revelation, and synthesize the compilation. A long-term longitudinal study, examining all key variables and their interactions, is warranted to fully understand trauma from severe TBI or DAI (36, 37). It should be acknowledged by authorities that caregivers themselves, in many ways, need to be cared for and rejuvenated through their being “rehabilitated” in some ways, to ensure they are always ready for the arduous task of care giving and rehabilitating their loved ones. Thus, ailing and healing of the mind deserves world recognition, validation and intervention**.**
